# High-risk HPV E5-induced cell fusion: a critical initiating event in the early stage of HPV-associated cervical cancer

**DOI:** 10.1186/1743-422X-7-238

**Published:** 2010-09-16

**Authors:** Peng Gao, Jie Zheng

**Affiliations:** 1 Department of Pathology and Pathophysiology, School of Medical Science, Southeast University, Dingjiaqiao Road, Nanjing 210009, PR China

## Abstract

**Background:**

Cervical cancer is strongly associated with high-risk human papillomavirus (HPV) and viral oncoproteins E5, E6 and E7 can transform cells by various mechanisms. It is proposed that oncogenic virus-induced cell fusion may contribute to oncogenesis if p53 or apoptosis is perturbed simultaneously. Recently, HPV-16 E5 was found to be necessary and sufficient for the formation of tetraploid cells, which are frequently found in precancerous cervical lesions and its formation is strongly associated with HPV state.

**Presentation of the hypothesis:**

We propose that high-risk HPV E5-induced cell fusion is a critical initiating event in the early stage of HPV-associated cervical cancer.

**Testing the hypothesis:**

Our hypothesis can be tested by comparing the likelihood for colony formation or tumorigenic ability in nude mice between normal HaCaT cells expressing all three oncogenic proteins and E5-induced bi-nucleated HaCaT cells expressing E6 and E7. Moreover, investigating premature chromosome condensation (PCC) in HPV-positive and negative precancerous cervical cells is another way to assess this hypothesis.

**Implication of the hypothesis:**

This viewpoint would change our understanding of the mechanisms by which HPV induces cervical cancer. According to this hypothesis, blocking E5-induced cell fusion is a promising way to prevent the progression of cervical cancer. Additionally, establishment of a role of cell fusion in cervical carcinogenesis is of reference value for understanding the pathogenesis of other virus-associated cancers.

## Background

Cervical cancer progression is strongly associated with infection of high-risk human papillomavirus (HPV) (e.g., HPV-16 and -18), which are detected in nearly all cervical cancers [1]. HPV is a small, nonenveloped DNA virus expressing three key oncoproteins: E5, E6 and E7, which possess the ability of transforming certain human cells *in vitro *and are considered to be associated with cervical carcinogenesis *in vivo *[2-5]. E6 and E7 are well known for their ability to inhibit the function of tumor suppressors p53 and pRb, respectively [6]. E5 has weak oncogenic properties which occur through increasing epidermal growth factor receptor (EGFR) and inhibiting the expression of major histocompatibility complex (MHC)-I and MHC-II on the plasma membrane [7]. Coexpression of E5 with either E6 or E7, however, promotes transformation by either oncoprotein alone [8].

Recently, the view that oncogenic virus-induced cell fusion may contribute to oncogenesis is appealing as all well-known human oncogenic viruses, including HPV, Hepatitis B virus, Hepatitis C virus, Epstein-Barr virus, Kaposi sarcoma virus and human T-lymphotropic virus type 1, have fusogenic activity [9-11]. Researches show that although most tetraploid cells resulting from non-oncogenic-induced cell fusion would undergo p53-dependent cell cycle arrest or apoptosis, they, however, survive and might be more prone to chromosomal instability (CIN) if p53 or apoptosis is perturbed [12,13]. It is notable that all oncogenic viruses mentioned above also possess proteins with these abilities [9]. As to HPV-associated cervical cancer, this mechanism may be operative as HPV-16 E5 was recently found to be necessary and sufficient for the formation of tetraploid cells [10,11], which are frequently found in precancerous cervical lesions and its formation is strongly associated with HPV state [14,15].

### Tetraploid cells in precancerous cervical lesions

Tetraploid cervical cells are often observed in precancerous lesions, and it has been established as a prognostic factor that allows to estimate the relative progression risk into more advanced lesions [15]. Moreover, aneuploid cells are frequently observed in these precancerous lesions. It is, therefore, proposed that a sequential pattern of chromosomal aberrations occurs during cervical carcinogenesis, where aneuploidy develops through chromosomal loss from a tetraploid intermediate. Another study investigated both tetraploid cervical cells and HPV state in precancerous cervical cells, results showed that tetraploid cervical cells were elevated in women diagnosed as either atypical squamous cells of undetermined significance (ASCUS)/HPV-positive or low-grade squamous intraepithelial lesion (LSIL)/HPV-positive as compared with normal/HPV-negative women, indicating that formation of tetraploid cells is obviously associated with HPV infection [14].

### HPV-16 E5 induces cell-cell fusion

HPV-16 E5 has all the characteristics of fusogenic proteins, including localization to the plasma membrane, high level of hydrophobicity, and the ability for dimmers [3]. Until recently, HPV-16 E5, however, has been identified to be necessary and sufficient to induce cell-cell fusion [10]. It is worth mentioning that HPV-16 E5 must be expressed on both cells for cell fusion to occur [11]. It is also found that tetraploid cells were produced with greater than a 3-fold frequency upon introduction of the HPV-16 genome into spontaneously immortalized human keratinocytes (HaCaT) as compared to cells transfected with an HPV-16 genome harboring a mutant E5 gene. By contrast, low-risk HPV-6b E5 could not induce cell fusion [10].

Based on the observations mentioned above, we hypothesize that high-risk HPV E5-induced cell fusion is a critical event in the early stage of HPV-associated cervical cancer.

## Presentation of the hypothesis

The fact that aneuploid cells are frequently observed in precancerous lesions with elevated proportion of tetraploid cells, the formation of which is obviously associated with HPV infection [14] suggests that formation of tetraploid cells is a critical event in cervical carcinogenesis, but the detail formation mechanism of tetraploidy is not clear. It is reported that expression of either HPV E6 or E7 alone is sufficient to deregulate cytokinesis and consequently produce tetraploid cells [16,17]. However, Hu et al. demonstrated that the formation of these cells is primarily attributed to E5 and E5-induced cell fusion, rather than E6, E7 and cytokinesis failure [10].

Tetraploid cells formed by accident can not undergo normal mitosis which would trigger p53-dependent cell cycle arrest or apoptosis [12,13], whereas, oncogenic virus-induced cell fusion is sufficient to induce CIN when fusion occurs concomitantly with expression of viral oncoproteins capable of perturbing p53 or apoptosis [12]. Consistently, although most tetraploid cells die out, whereas, coexpression of HPV-16 E6/E7 enhances the proliferation of these cells and the likelihood for colony formation elevates 3-fold [10].

Based on the observations mentioned above, we propose that high-risk HPV E5-induced cell fusion may play a critical role in the early stage of HPV-associated cervical cancer. However, it is widely accepted that increasingly deregulated expression of the E6-E7 oncogenes of high-risk HPVs has been identified as the major transforming factor in the pathogenesis of cervical dysplasia and derived cancers [2]. In fact, these two mechanisms are not mutually exclusive as E5 functions only in the early stage, whereas, E6 and E7 act throughout the carcinogenesis. Additionally, it is notable that expression levels of all three oncoproteins in host cell are low for tight restriction in the early stage, and expression of E5 is nearly not detectable in all late stages for integration [3]. Moreover, the ability of E6 and E7 to transform various cells have been demonstrated only in overexpression systems, therefore, whether it also occurs in natural settings is not known. We also notice that *in vitro *[18,19] and clinic studies [20] reveal that chromosomal instability and aneuploidization seem to precede and favor integration of HPV genomes, which in turn leads to expression of viral-cellular fusion transcripts and further enhances expression of the E6-E7 genes, which renders transformed cells strong growth advantages [21].

## Testing the hypothesis

In order to determine whether high-risk HPV E5-induced cell fusion is important for initial transformation, we can compare the likelihood for colony formation or tumorigenic ability in nude mice between normal HaCaT cells expressing all three oncogenic proteins and E5-induced bi-nucleated HaCaT cells expressing E6 and E7. In our hypothesis, cell fusion and cell cycle deregulation are two key events for initiation of transformation. Therefore, inhibition of cell fusion should significantly decrease the likelihood for transformation. Moreover, investigating premature chromosome condensation (PCC) in HPV-positive and negative precancerous cells is another way to test this hypothesis. PCC refers to condensation of interphase chromosomes following fusion between an interphase and a mitotic cell [22], and it can be used as a tool to detect the existence of cell fusion [23].

## Implication of the hypothesis

Understanding the cause of cancer is critical for effective diagnosis, prevention and therapy. The recent view that human oncogenic virus-induced cell fusion can initiate cancer is an appealing mechanism. In this article, we propose that high-risk HPV E5-induced cell fusion is an important event in the early stage of HPV-associated cervical cancer. This viewpoint will change our understanding of the mechanisms by which HPV induces cervical cancer. According to this hypothesis, blocking HPV E5-induced cell fusion is a promising way to prevent the progression of cervical cancer. Additionally, establishment of a role of cell fusion in carcinogenesis of cervical cancer is of reference value for understanding the pathogenesis of other virus-associated cancers.

## Competing interests

The authors declare that they have no competing interests.

## Authors' contributions

Both authors contributed equally to this manuscript. Both authors read and approved the final manuscript.

## References

[B1] zur Hausen H Papillomavirus infections--a major cause of human cancers Biochim Biophys Acta 1996 1288 F55 78 8876633 10.1016/0304-419x(96)00020-0

[B2] Liu Y Chen JJ Gao Q Dalal S Hong Y Mansur CP Band V Androphy EJ Multiple functions of human papillomavirus type 16 E6 contribute to the immortalization of mammary epithelial cells J Virol 1999 73 7297 7307 10438818 10.1128/jvi.73.9.7297-7307.1999 PMC104255

[B3] Zerfass K Schulze A Spitkovsky D Friedman V Henglein B Jansen-Dürr P Sequential activation of cyclin E and cyclin A gene expression by human papillomavirus type 16 E7 through sequences necessary for transformation J Virol 1995 69 6389 6399 7666540 10.1128/jvi.69.10.6389-6399.1995 PMC189538

[B4] Wazer DE Liu XL Chu Q Gao Q Band V Immortalization of distinct human mammary epithelial cell types by human papilloma virus 16 E6 or E7 Proc Natl Acad Sci USA 1995 92 3687 3691 10.1073/pnas.92.9.3687 7537374 PMC42026

[B5] Leechanachai P Banks L Moreau F Matlashewski G The E5 gene from human papillomavirus type 16 is an oncogene which enhances growth factor-mediated signal transduction to the nucleus Oncogene 1992 7 19 25 1311062

[B6] Narisawa-Saito M Kiyono T Basic mechanisms of high-risk human papillomavirus-induced carcinogenesis: roles of E6 and E7 proteins Cancer Sci 2007 98 1505 1511 10.1111/j.1349-7006.2007.00546.x 17645777 PMC11158331

[B7] Tsai TC Chen SL The biochemical and biological functions of human papillomavirus type 16 E5 protein Arch Virol 2003 148 1445 1453 10.1007/s00705-003-0111-z 12898324

[B8] Stöppler MC Straight SW Tsao G Schlegel R McCance DJ The E5 gene of HPV-16 enhances keratinocyte immortalization by full-length DNA Virology 1996 223 251 254 10.1006/viro.1996.0475 8806560

[B9] Duelli D Lazebnik Y Cell-to-cell fusion as a link between viruses and cancer Nat Rev Cancer 2007 7 968 976 10.1038/nrc2272 18034186

[B10] Hu L Plafker K Vorozhko V Zuna RE Hanigan MH Gorbsky GJ Plafker SM Angeletti PC Ceresa BP Human papillomavirus 16 E5 induces bi-nucleated cell formation by cell-cell fusion Virology 2009 384 125 134 10.1016/j.virol.2008.10.011 19041112 PMC2658674

[B11] Hu L Ceresa BP Characterization of the plasma membrane localization and orientation of HPV16 E5 for cell-cell fusion Virology 2009 393 135 143 10.1016/j.virol.2009.07.034 19712955 PMC2753727

[B12] Duelli DM Padilla-Nash HM Berman D Murphy KM Ried T Lazebnik Y A virus causes cancer by inducing massive chromosomal instability through cell fusion Curr Biol 2007 17 431 437 10.1016/j.cub.2007.01.049 17320392

[B13] Andreassen PR Lohez OD Lacroix FB Margolis RL Tetraploid state induces p53-dependent arrest of nontransformed mammalian cells in G1 Mol Biol Cell 2001 12 1315 1328 11359924 10.1091/mbc.12.5.1315 PMC34586

[B14] Olaharski AJ Eastmond DA Elevated levels of tetraploid cervical cells in human papillomavirus-positive Papanicolaou smears diagnosed as atypical squamous cells of undetermined significance Cancer 2004 102 192 199 10.1002/cncr.20259 15211479

[B15] Olaharski AJ Sotelo R Solorza-Luna G Gonsebatt ME Guzman P Mohar A Eastmond DA Tetraploidy and chromosomal instability are early events during cervical carcinogenesis Carcinogenesis 2006 27 337 343 10.1093/carcin/bgi218 16123119

[B16] Incassati A Patel D McCance DJ Induction of tetraploidy through loss of p53 and upregulation of Plk1 by human papillomavirus type-16 E6 Oncogene 2006 25 2444 2451 10.1038/sj.onc.1209276 16369493

[B17] Heilman SA Nordberg JJ Liu Y Sluder G Chen JJ Abrogation of the postmitotic checkpoint contributes to polyploidization in human papillomavirus E7-expressing cells J Virol 2009 83 2756 2764 10.1128/JVI.02149-08 19129456 PMC2648292

[B18] Duensing S Duensing A Flores ER Do A Lambert PF Münger K Centrosome abnormalities and genomic instability by episomal expression of human papillomavirus type 16 in raft cultures of human keratinocytes J Virol 2001 75 7712 7716 10.1128/JVI.75.16.7712-7716.2001 11462043 PMC115006

[B19] Duensing S Duensing A Crum CP Münger K Human papillomavirus type 16 E7 oncoprotein-induced abnormal centrosome synthesis is an early event in the evolving malignant phenotype Cancer Res 2001 61 2356 2360 11289095

[B20] Melsheimer P Vinokurova S Wentzensen N Bastert G von Knebel Doeberitz M DNA aneuploidy and integration of human papillomavirus type 16 e6/e7 oncogenes in intraepithelial neoplasia and invasive squamous cell carcinoma of the cervix uteri Clin Cancer Res 2004 10 3059 3063 10.1158/1078-0432.CCR-03-0565 15131043

[B21] Ziegert C Wentzensen N Vinokurova S Kisseljov F Einenkel J Hoeckel M von Knebel Doeberitz M A comprehensive analysis of HPV integration loci in anogenital lesions combining transcript and genome-based amplification techniques Oncogene 2003 22 3977 3984 10.1038/sj.onc.1206629 12813471

[B22] Johnson RT Rao PN Mammalian cell fusion: induction of premature chromosome condensation in interphase nuclei Nature 1970 226 717 722 10.1038/226717a0 5443247

[B23] Kovacs G Premature chromosome condensation: evidence for in vivo cell fusion in human malignant tumours Int J Cancer 1985 36 637 641 10.1002/ijc.2910360602 4066070

